# Smallholder farmers’ knowledge and willingness to pay for insect-based feeds in Kenya

**DOI:** 10.1371/journal.pone.0230552

**Published:** 2020-03-25

**Authors:** Shaphan Y. Chia, John Macharia, Gracious M. Diiro, Menale Kassie, Sunday Ekesi, Joop J. A. van Loon, Marcel Dicke, Chrysantus M. Tanga

**Affiliations:** 1 Laboratory of Entomology, Wageningen University & Research, Wageningen, the Netherlands; 2 International Centre of Insect Physiology and Ecology *(icipe)*, Nairobi, Kenya; University of Florida, UNITED STATES

## Abstract

Edible insects are increasingly being considered as sustainable alternatives to fish and soybean meals in animal feed because of their high nutritional quality and environmental benefits. However, successful introduction of a new product to the market depends on the target user’s acceptance. Thus, evaluating the potential demand of insect-based feeds would provide relevant information for policy development. The present study assessed farmers’ knowledge on edible insects as feed, their acceptance of integrating insect meals in animal feeds and willingness to pay (WTP) for insect-based feed (IBF) using a contingent valuation method. A household survey was conducted among 957 randomly selected farmers including: 409 poultry, 241 fish and 307 pig farmers in four counties in Kenya. Results of the study reveal that over 70 and 80% of poultry and fish farmers, respectively, are aware that insects can be used as a feed ingredient. In addition, over 60 and 75% of poultry and fish farmers, respectively, consider insects as a good component of feed. Poultry, pig and fish farmers interviewed accepted and showed willingness to pay for IBF. Regression analysis indicated that age, gender, education, marital status, distance to feed trader, awareness of insects as feed, attitude towards insects, acceptance of insect species, availability of agricultural inputs, use of commercial feeds, availability of training and market information had a significant influence on the WTP for IBF. Therefore, increased extension services to educate famers on the nutritional benefits of insect meals in animal feeds and existing market opportunities are expected to improve farmers’ attitude towards utilization and consequently enhance WTP for IBF, which in return would significantly reduce the existing pressure on conventional fishmeal feed resources. Our findings provide the first insights into the market opportunities of including insect meals in the animal feed value chain in Kenya.

## Introduction

In livestock and aquaculture production, feed is the most important input, representing 60–70% and 40–80% of total cost of production, respectively [1–3]. Feed production requires high resource inputs and the current food-feed competition as well as overfishing represent major sustainability issues that need viable solutions. Global demand of feed is increasing and projection by 2050 revealed that over a billion tonnes of cereals will be required in animal feed as opposed to about eight hundred million tonnes currently used. Developing countries will likely experience most of the increase in demand of animal feed [1,4]. Livestock and aquaculture production provide employment, income generation and food security opportunities especially in vulnerable communities [5–8].

The livestock sector, including poultry and pig production among other livestock species contributes about 42% of the Kenya’s agricultural Gross Domestic Product (GDP), 12% of the national GDP, 30% of total marketed agricultural products and employs about 50% of the agricultural sector labour force [9,10]. Kenya’s poultry population is estimated at 31 billion birds, 75% of which are indigenous chicken, 22% are broilers and layers [11]. The sector produces about 605,000 metric tonnes of meat annually. In pig production, smallholder farms keep 5–100 pigs and make up 70% of the total pig producers. Feed costs alone represent up to 80% of total costs of production [12]. The Kenyan fisheries and aquaculture sector employ about 20,000 people [13, 14]. Kenya is the fourth largest producer of freshwater fish in Africa. However, several factors including lack of market information, low levels of extension services and inadequate availability of quality and affordable feeds prevent the sector from realizing its full potential [15, 16].

In Kenya, major poultry feed categories include chick mash, growers’ mash, layers’ mash, broilers’ mash and Kienyeji mash. Pigs are fed with pig starter, creep pellet, sow and weaner and pig finisher feeds. Fish feeds include floating pellets and mash feed. In these feeds, fishmeal and soybean meal are the major protein ingredients. However, reduced availability, high cost and environmental implications of exploiting these resources represent major constraints to achieving optimal production, especially for smallholder producers in the developing countries [17–21]. In view of the above concerns, researchers, policy makers, private and public institutions including the Food and Agricultural Organization (FAO) have called for diversification and innovation towards sustainable feed protein sources such as edible insects [22–24].

Edible insects have traditionally been part of livestock diets especially in the tropics and may provide an alternative source of protein and other nutrients in livestock and aquaculture feeds [24–26]. Insects contain valuable proteins with well-balanced amino acid profiles, fats with rich fatty acid contents as well as micronutrients. The use of insects as an alternative protein source is advantageous because they can be sustainably mass reared on various streams [27, 28]. The black soldier fly (BSF) *Hermetia illucens* L. (1758) (Diptera: Stratiomyidae) and the synanthropic housefly *Musca domestica* L. (Diptera: Muscidae) for example, feed on organic side streams and produce nutrient-rich larvae that could be used as ingredients in animal feeds while helping to reduce waste on which the larvae are reared [23,29]. Insects contain 40–60% protein on a dry matter basis and have been found to be a suitable alternative to fishmeal and soybean meal in animal feed. Furthermore, insects release smaller amounts of greenhouse gases per unit of protein produced than cattle, pigs and chickens [4, 26, 30, 31].

Feed manufacturers are willing to include insects in their feed formulation, given favourable legislation and marketplace acceptance [32]. However, little is known about farmers’ perception towards the use of insects in animal feed. Such perception may affect the success of introducing insect-based feed (IBF), as well as the consumer acceptance of products resulting from animals fed IBF [33]. So far, only a few studies have documented consumer acceptance of insects as feed, all in European countries including Belgium [33], France [34], Germany [35], Poland [36], Italy [37, 38] and the United Kingdom [39]. Overall, these studies found a favourable attitude and willingness to accept insects in animal feed and resulting products from animals fed with IBF among respondents. Furthermore, consumer willingness to pay (WTP) for insects and insect-based products for human consumption has been assessed and results show that consumers who are familiar with the idea of insects as food are more likely to accept insect-based feeds. In addition, consumers are willing to accept insect-based products with high nutritional quality. Therefore, information campaigns and identifying suitable target markets are crucial for promoting a new product [40–43].

While the findings from these previous studies are useful, such information is limited or lacking for Africa, particularly for smallholder farmers who make up 70% of all the producers. The present study aims to provide the first insights into farmers’ knowledge on the use of insects as ingredients in animal feed and the potential demand for IBF for fish and livestock using household level data in major poultry, fish and pig producing counties of Kenya. Although, the use of insects for feeding farmed animals represents a promising alternative to the expensive fishmeal because of the nutritional properties of insects and the possible environmental benefits, given the sustainability of this type of farming, there is a lack of consensus among livestock and aquaculture farmers in Kenya on the use of insect-based feeds and social acceptance of this practice. The novelty of the current research work also includes the use a double-bound discrete choice model to investigate livestock and aquaculture producers’ willingness to pay for insect-based feeds for their farm animals. The results generated from this study strongly support the need for insect-based meal inclusion in feeds following standard regulation practices. However, it is worth noting that the successful introduction of a new product in the market depends on the product’s marketplace acceptance by the target users, which ultimately will affect the WTP for the product [27, 44–46]. Therefore, we evaluated knowledge, attitudes, practices and WTP for IBF among poultry, fish and pig farmers in four counties in Kenya across different agro-ecological zones.

### Theoretical framework

Producers are in a constant search for new technologies or inputs with novel attributes to reduce production costs and increase revenues. However, these products do not have an existing market, making it hard to estimate their demand potential. As a result, the producer demand estimation relies on stated acceptance. One of the stated-preference methods used to elicit demand is known as the contingent valuation method (CVM) [47]. The CVM is a non-market valuation method used to find the economic value of non-market commodities. It uses hypothetical survey questions in order to elicit peoples’ acceptance of public goods. It is used to find out what the people are willing to pay for specified improvements in the goods. In CVM, absence of markets is circumvented by presenting the consumers/producers with hypothetical markets where they can be provided with information about the products and then asked how much they are willing to pay to obtain the good described. There are four commonly utilized elicitation formats in CVM: Open-ended, Dichotomous Choice, Payment Card, and Bidding Game [47]. The bidding game was used in this study. It involves a series of yes/no questions aimed at finding the maximum willingness to pay. The repeated nature of this technique allows a greater amount of time for the respondent to scrutinize their response, and thus gives results that have greater construct validity. Elicitation of contingent valuation employs either of two methods: single or double bounded contingent valuation method.

In the single bound model, the respondents are faced with a single bid value to which their response is either a “yes” or “no” [47]. “Yes” denotes WTP the proposed amount while “no” denotes refusal to pay the proposed amount. Alternatively, they can be assessed on the likelihood of paying for the product without attaching any price to it. The probability of obtaining either a “yes” or “no” response can be written as follows:

$$Prob\left(no\right)=\pi^{n}=G\left(BID;\;\theta \right)$$

$$Prob\left(yes\right)=\pi^{y}=1-G\left(BID;\;\theta \right),$$

Where *G* (*BID; θ*) is the statistical distribution function with parameter θ, which can be estimated using a logit or probit model, a qualitative choice model. Logit or probit model for a single bid value can be expressed in two forms; log-logistic or the logistic cumulative distribution.

The log-logistic cumulative distribution is expressed as follows:

$$G\left(Bid\right)=\frac{1}{\left[1+e^{a-b\left(ln\;Bid\right)}\right]}$$

The logistic cumulative distribution is expressed as follows:

$$G\left(Bid\right)=\frac{1}{\left[1+e^{a-b\left(Bid\right)}\right]}$$

Where θ = (*a*, *b*), *a* and *b* are the intercept and slope coefficients to be estimated. The statistical model can be interpreted to mean that an individual whose aim is to maximize utility within a random utility context will say “yes” to a *BID* only if the *BID* is less than or equal to his maximum WTP and will say “no” if the *BID* is greater. Alternatively, for a case that has no bid value attached to the model, the probability of obtaining either a “yes” or “no” response can be written as follows:

$$Prob\left(no\right)=\pi^{n}=G\left(X;\;\theta \right)$$

$$Prob\left(yes\right)=\pi^{y}=1-G\left(X;\;\theta \right),$$

Where *X* represents the control, variables used in the model [48].

In a double-bound model, the respondents are faced with a two-sequence-bid offer. In the first offer, they are asked whether they will accept or reject the bid, then the second bid is offered depending on the respondent’s first bid response, a higher bid if the response was yes and a lower bid if the response was no. This results in four possible responses: (1) both answers are “yes”, (2) both answers are “no”, (3) a “yes” followed by a “no” and 4) a “no” followed by a “yes” [49].

This two-sequence-bid provides a bound of the respondent’s WTP. The WTP is right censored if the answer to the initial and higher bids is “yes” and left censored if the response to the first and second bids is “no”. If both answers are alternate of yes and no, then their WTP is intermediate with the second bid acting as an upper or lower bid. The likelihood of these outcomes is as shown in S1 Fig.

It is assumed that a respondent’s maximum WTP is lower than or equal to the lowest bid ($\max WTP<Bid_{i}^{L}$) if he or she rejects the first and second (lower) bid offers. It is assumed that the respondent’s maximum WTP lies between the lower and the first bid offer ($Bid_{i}^{L}\leq \text{maxWTP}<$ *Bid*_*i*_) if the respondent rejects the first bid but accepts the second lower bid offer. If the respondent is willing to accept the first bid but rejects the second higher bid offer, it is assumed that the respondent’s maximum WTP lies between the second higher and the first bid offers ($Bid_{i}^{H}>\text{maxWTP}>Bid_{i}$). Finally, if the respondent accepts the first and second higher bids, then it is assumed that the respondent’s maximum WTP is greater than or equal to the second higher bid offer ($\text{maxWTP}\geq Bid_{i}^{H}$).

The double bound dichotomous choice model improves on the single bound dichotomous choice model by providing a two-level bidding process [48]. In this study a double-bound logit model was used to estimate WTP and the factors that influence WTP for IBF among the farmers. A positive correlation between a variable and WTP means that an increase in the variable leads to an increase in the probability of WTP for IBF. Furthermore, a negative correlation with WTP means that an increase in the variable leads to a decrease in the probability of WTP for IBF.

## Materials and methods

### Study area and data collection

**Fig 1:**
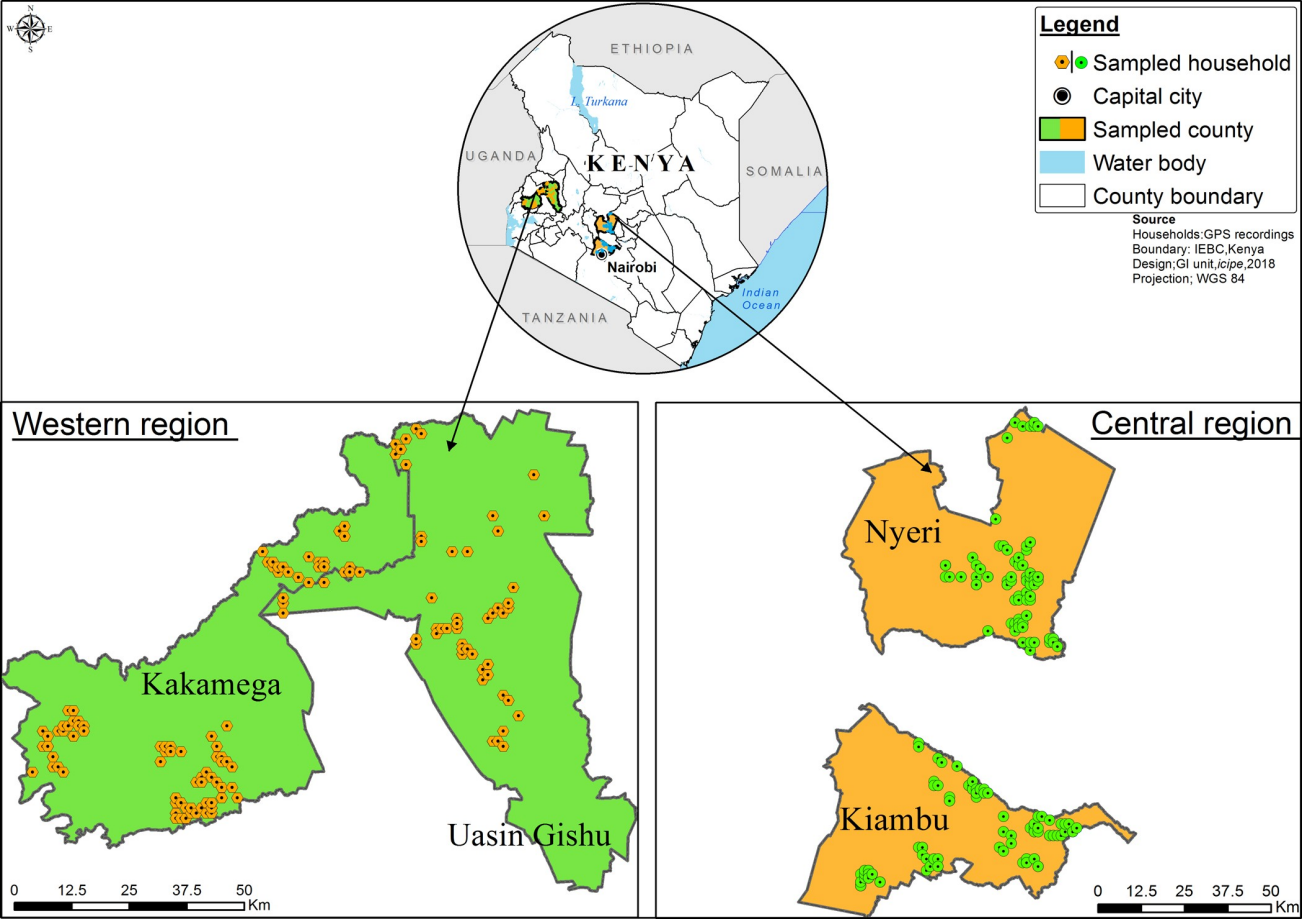
Map representing the four study areas (counties) in two geographical regions of Kenya. Green colour represents counties sampled in the Western region, orange represents counties sampled in the Central region of Kenya.

This study was conducted in four counties in Kenya, including Kiambu, Nyeri, Kakamega and Uasin Gishu (Fig 1). A purposive sampling method was employed to select sub-counties in each of four counties, based on the production statistics of the three animal types including pig, poultry and fish. Respondents within each sub-county were randomly selected. The sample frame composed of a census of active smallholder pig farmers, poultry farmers and fish farmers in the survey sites compiled by the respective sub-county agricultural officers for these. A total of 957 farmers were interviewed in the four Counties (723 farmers with a single enterprise, 196 farmers with two enterprises and 38 farmers with all three enterprises). In total 409 poultry famers were interviewed, distributed as follows: Kiambu (79), Nyeri (89), Kakamega (98) and Uasin Gishu (143). A total of 307 pig farmers were interviewed: Kiambu (102), Nyeri (63), Kakamega (96) and Uasin Gishu (46). A total of 241 fish farmers were interviewed: Kiambu (29), Nyeri (68), Kakamega (75) and Uasin Gishu (69). Data were collected at the household level by trained enumerators using CSPro version 7.0, data collection software addressing the following aspects: socioeconomic characteristics of the respondents, their knowledge, attitudes, practices and acceptance of different insect species, availability of agricultural support services, feed use and distance to feed market (trader). This study was approved by the international centre of insect physiology and ecology (*icipe*) science committee, which is the institutional body under the board of the centre. In addition, our questionnaire had an introductory statement that sought the respondent’s consent to participate in the survey. Farmers’ WTP for IBF and prices that farmers were willing to offer per unit of IBF, availability of market and financial institutions were also assessed.

### Empirical model

We model a farmer’s WTP for IBF using a modified single-equation logit model of the form [50]:

$$Y_{i}=\beta_{O}+\beta_{i}X_{i}+\varepsilon_{i}$$

Where *Y* is the outcome variable for farmers’ WTP having the bids 1 and 2 values and their responses (having bids as continuous values and the value of one if farmers are willing to pay for IBF in bid 1 or 2 and zero if the farmers are not willing to pay for IBF in bid 1 or 2), *i* indexes individual farmer’s WTP, *β*_*0*_ is the intercept, *β*_*i*_ is the regression coefficient, *X* is a vector of explanatory variables that affect farmers’ WTP, *ɛ*_*j*_ is an error term, which assumes a normal distribution (mean = 0, variance = 1). Selection of explanatory variables used in this study (Table 1) were guided by a review of theoretical and empirical studies on the determinants of WTP for agricultural products [38, 41–44].

**Table 1:** Description of variables and their expected signs.

Variable		Description	Expected Sign
Dependent variable	WTP		
	Bid 1	The First BID offered to the respondents	
	Bid 2	The Second BID offered to the respondent	
	Answer 1	The respondent to the First BID	
	Answer 2	The Response to the Second BID	
	Gender	Gender of the household head	+/-
Independent variables	Age	Age of the household head	+/-
	Age Squared	Square of the Age	-
	Education level	Education level of the Household head	+/-
	Marital status	Marital Status of the household head	+/-
	Household size	Number of persons in the house	+/-
	Income	Income of the household	+
	Commercial feed	Type of feed used commercial or otherwise	-
	Distance	Distance to the nearest feed trader	-
	Make own feed	Does the household make their own livestock feed	+/-
	Number of growers owned	Number of growers owned	+
	Number of chicks owned	Number of chicks owned	+/-
Preferred Insects and use	Aware that poultry feed on insect	Aware that poultry feed on insect	+
	Insect good source of poultry feed	Is insect a good source of poultry feed	+
	Ever used insect as feed	Ever used insect as feed	+
	Preference Score	The number of insects preferred	+
	Availability of microcredit	Availability of microcredit	+
Availability of agricultural inputs, technologies and credit	Availability of extension	Availability of extension	+
	Availability of training	Availability of training	+
	Availability of agricultural inputs	Availability of agricultural inputs	+/-
	Availability of treatment	Availability of treatment	+
	Availability of market information	Availability of market information	+
Region	Nyeri	1 = Nyeri, 0 = Otherwise	+/-
	Kiambu	1 = Kiambu, 0 = Otherwise	+/-
	Kakamega	1 = Kakamega, 0 = Otherwise	+/-
	Uasin Gishu	1 = Uasin Gishu, 0 = Otherwise	+/-

**Gender:** Gender is represented by a dummy variable, which takes a value of 1 if the household is male and 0 if female. According to literature, female-headed households have less access to improved technologies, land and extension than male-headed household [51]. Therefore, the study hypothesized that the male-headed households have a better WTP for insect-based feeds [52].

**Age** is captured as a continuous number of years of the household head. The age of farmer is expected to have a positive effect on WTP for insect-based feed because the accumulated experience of older farmers helps them to make early willing to pay decision [52].

**Age squared** is used to measure the effect of experience on the WTP. Experience in farming is hypothesized to have a positive influence on the WTP [53].

**Education level** of the household head was measured by the number of years of formal education. According to literature, household heads with higher levels of education are expected to show higher levels of WTP, as they might have been exposed thus better access to information. Moreover, education has been known to enable farmers access new information and ideas [52]. It is hypothesized that education of household head has a positive impact WTP for insect-based feeds.

**Household size:** In agricultural production human labor is a key operator. Therefore, a large family size may indicate that there is labor availability. Thus, a farm with larger number of workers (whether hired or family in terms of man-equivalent) is hypothesized to be more likely to buy new technologies especially where there is increased labor demand for production [52]. Results from other researchers also support this hypothesis [54].

**Income** is a continuous variable that measures the proceeds from either crop, livestock or both crops and livestock enterprises in a particular year. According to literature, the higher the cash income, the greater the capacity of the household to buy new technology [55]. This variable is hypothesized to influence positively WTP of the households. Other findings also support this hypothesis [56].

**Commercial feed** use is a dummy variable that measures the existing practice of the household on feed usage. A household that is already using commercial feeds in the production are likely to continue purchasing the insect-based enriched feeds as they will expect high production when considering the feeds as better and improved. Thus, this variable is hypothesized to positively influence WTP [57].

**Distance to the feed market** is a continuous variable that is measured in walking minutes. This variable is hypothesized to negatively influence WTP as it affects the timely input delivery and output disposal [52,58, 59].

**Make own feed** is a dummy variable with 1 denoting those households that make their own feed and 0 those who do not. Farmers that made their own feed will find it hard to incorporate the new ingredients as they imply change of mixing ratios and sourcing of the ingredients. Therefore, the study hypothesized that this variable will have a negative influence on the household’s WTP [57].

**Number of animals (poultry, fish and pig)** determine the risk prevalent on the failure of the new technology to perform as expected. Farmers with many animals at the delicate stage of growth such as chicks who purely depend on the specially mixed feeds will not be quick to adopt new technology thus negatively influencing their WTP. On the other hand, having many animals provide a reason for seeking to improve their production thus may result in the adoption of new technologies that improve production [60]. Therefore, this variable was hypothesized to influence WTP either positively or negatively depending on the farmer status and level of production.

**Preference score** are continuous variable, showing the number of insects preferred for use as ingredients in livestock and aquaculture feed. Farmers who prefer any of the stated insects for use in animal feed are most likely to adopt and thus buy insect-based feeds [52]. Therefore, these variables were hypothesized to either positively or negatively impact the households WTP.

**Availability of agricultural inputs, technologies and credit** variables are dummies with 1 denoting availability and 0 non-availability. Literature shows that availability of agricultural inputs, technologies and credit helps farmers to access, to be aware of the new knowledge and to be able to access skill to improve their productivity [52]. Thus, in this study availability of agricultural inputs, technologies and credit are expected to influence WTP for insect-based feeds decisions positively [61].

**Training** is a dummy variable with 1 denoting availability of training and 0 denoting non-availability. Training is hypothesized to positively influence WTP as it provides the necessary information on the benefits of the new technology and how to effectively apply it [52, 62].

**Region** variables control for the regional effects with their coefficient hypothesized to either negatively or positively influence the WTP [63].

We estimate the empirical model for several categories of poultry, pig, and fish farmers based on the expectation that factors associated with WTP for insect-based feed will vary across farmer type. Poultry farmers were categorized into those rearing Kienyeji (an indigenous type of chicken), Layers (laying chickens aged 19–76 weeks), Growers (chickens aged 8–18 weeks), and Chicks (young birds aged 0–8 weeks). Fish farmers were grouped according to type of feed currently used: floating pellets (finely ground feed that has been compressed and molded into pellets in a pellet mill and float on the surface of water when served to grower and finisher fish stages) and feed mash (a finely ground feed formulated and used in moist form for farmed juvenile fish). Pig farmers included those raising finisher (pigs weighing over 55 kg) or sows and weaners (pigs up to 55 kg) and adult breeding pigs.

## Results

### Demographic characteristics of the study population

A total of 957 farmers participated in this study. Fifty nine percent (59%) of poultry, 34% of fish and 49% of pig farmers were females (Table 2). Mean age varied significantly for male and female respondents in poultry, fish and pig production. Fish farmers had the highest (52.5 and 49.2 years) mean age while pig farmers had the lowest (48.5 and 45.7 years) mean age for male and female respondents, respectively. For all categories (poultry, fish and pig), mean number of years of education differed significantly for male and female respondents; male farmers were more educated than the females (Table 2). Household size and distance to feed trader were similar for male- and female-headed households for poultry, fish and pig farmers. There were on average, five members per household (Table 2) across the study locations.

**Table 2:** Demographic characteristics of the farmer populations.

Parameter	Range	Poultry (N = 409)			Fish (N = 241)			Pig (N = 307)		
		Male N (%)	Female N (%)	t-value	Male N (%)	Female N (%)	t-value	Male N (%)	Female N (%)	t-value
Age (years)	18–30	13 (3.2)	21 (5.1)		14 (5.8)	7 (2.9)		21 (6.8)	21 (6.8)	
	31–40	20 (4.9)	57 (13.9)		19 (7.9)	12 (5.0)		29 (9.5)	38 (12.4)	
	41–50	42 (10.3)	67 (16.4)		38 (15.8)	26 (10.8)		34 (11.1)	38 (12.4)	
	>50	91 (22.3)	98 (24.0)		89 (36.9)	36 (14.9)		72 (23.5)	54 (17.6)	
	Sub-total	166 (40.6)	243 (59.4)		160 (66.4)	81 (33.6)		156 (50.8)	151 (49.2)	
	Mean age	51.24	47.23	-3.12**	52.47	49.23	-1.70	48.48	45.74	-1.68
Education (years)	No formal	2 (0.5)	3 (0.7)		2 (0.8)	3 (1.2)		1 (0.3)	6 (2.0)	
	Primary	45 (11)	98 (24.0)		60 (24.9)	32 (13.3)		47 (15.3)	68 (22.2)	
	Secondary	62 (15.2)	93 (22.7)		50 (20.8)	34 (14.1)		68 (22.2)	57 (18.6)	
	Tertiary	57 (13.9)	49 (12.0)		48 (19.9)	12 (5.0)		40 (13.0)	20 (6.5)	
	Mean duration	11.38	10.09	-3.64**	10.83	9.70	-2.24*	10.96	9.46	-3.75**
Household size	1–4	77 (18.8)	93 (22.7)		73 (30.1)	32 (13.3)		79 (25.7)	54 (17.6)	
	5–8	73 (17.9)	127 (31.1)		73 (30.1)	42 (17.4)		65 (21.2)	83 (27.0)	
	>8	16 (3.9)	23 (5.6)		14 (5.8)	7 (2.9)		12 (3.9)	12 (3.9)	
	Mean	5.27	5.40	0.51	5.19	5.26	0.19	4.94	5.19	0.92
Distance to feed trader (Km)	0.01–15	28 (6.9)	80 (19.6)		26 (10.8)	11 (4.6)		21 (6.8)	16 (5.2)	
	16–30	4 (1.0)	7 (1.7)		4 (1.7)	2 (0.8)		13 (4.2)	8 (2.6)	
	31–45	22 (5.4)	12 (2.9)		2 (0.8)	2 (0.8)		8 (2.6)	5 (1.6)	
	>45	19 (4.7)	26 (6.4)		6 (2.5)	3 (1.2)		4 (1.3)	5 (1.6)	
	Mean	29.54	35.5	0.57	41.81	53.47	0.35	23.67	32.35	1.09

Significance levels:

** P < 0.01,

* P < 0.05, t-test. Values in parentheses represent percentage of male or female within a given age range.

### Farmer knowledge, attitudes and practices towards insects as an alternative source of feed for poultry, fish and pigs

The proportion of female farmers who were aware that insects can be used as feed for poultry was significantly higher than for the males. Male and female fish farmers were similarly aware that insects can be used as feed for fish (Table 3). A significantly higher proportion of female poultry farmers had a positive attitude towards insects as feed than the males. There was no significant difference between proportions of male and female fish farmers with regards to their attitude toward the use of insects in animal feeds. However, only a small proportion of both poultry and fish farmers demonstrated that they had previously used insects to feed their animals (25–38% of respondents). The proportion of male famers who previously used insects as feed for their fish was significantly higher than for female fish farmers. It was common to find poultry, fish and pig farmers engaged in the practice of making their own feed as well as using commercial feeds. Feed items (conventional feed) frequently used by the smallholder farmers included: vegetables, grains, food remains. On average, less than 20% of male and female respondents made their own feed in all animal categories (Table 3). More male than female pig farmers made their own feed while more female poultry farmers than males used conventional feeds (Table 3).

**Table 3:** Farmers’ knowledge, attitude and practices towards insects as an alternative source of feed for poultry, fish and pigs.

Parameter	Description	Poultry (n = 409)			Fish (n = 241)			Pig (n = 307)		
		Male (n = 166)	Female (n = 243)	z-value	Male (n = 160)	Female (n = 81)	z-value	Male (n = 156)	Female (n = 151)	z-value
**Knowledge**	Aware that insects can be used as feed (%)	60	77	3.75**	83	74	-1.54	-	-	-
**Attitudes**	Insects are a good source of feed (%)	55	68	2.77**	79	75	-0.72	-	-	-
**Practices**	Make their own feed (%)	11	8	-1.24	20	16	-0.74	21	9	-2.95**
	Ever used insects as feed (%)	31	31	0.12	38	25	-2.09*	-	-	-
	Used commercial feeds (%)	81	80	-0.22	74	69	-0.76	69	67	-0.32
	Used conventional feeds (%)	67	85	4.37**	68	67	-0.23	85	82	-0.74

Significance levels:

*** P < 0.01,

** P < 0.05,

* P<0.1, z-test. (-) Not evaluated. Conventional feed = vegetables, grains and food remain used as feed.

### Acceptance of insect species and availability of agricultural support services and inputs

**Fig 2:**
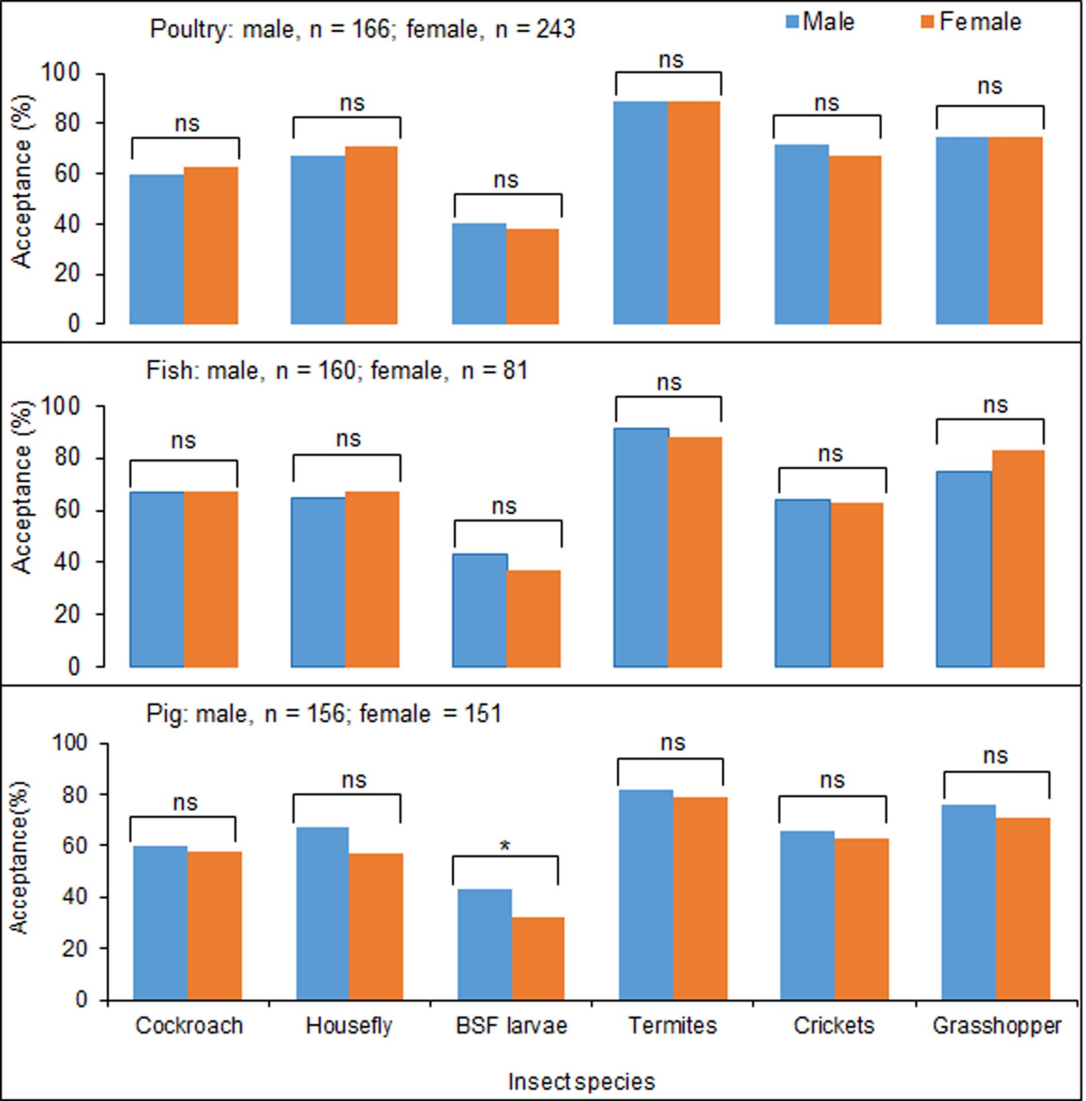
Percentage farmers that accept insects as feed ingredients among poultry, fish and pig farmers. Bars with an asterisk are significantly different for male and female respondents, P < 0.05, two-proportion z-test. Bars with “ns” are not significantly different for male and female respondents, P < 0.05, two-proportion z-test. BSF = black soldier fly. For additional statistical analyses see Table 3.

**Fig 3:**
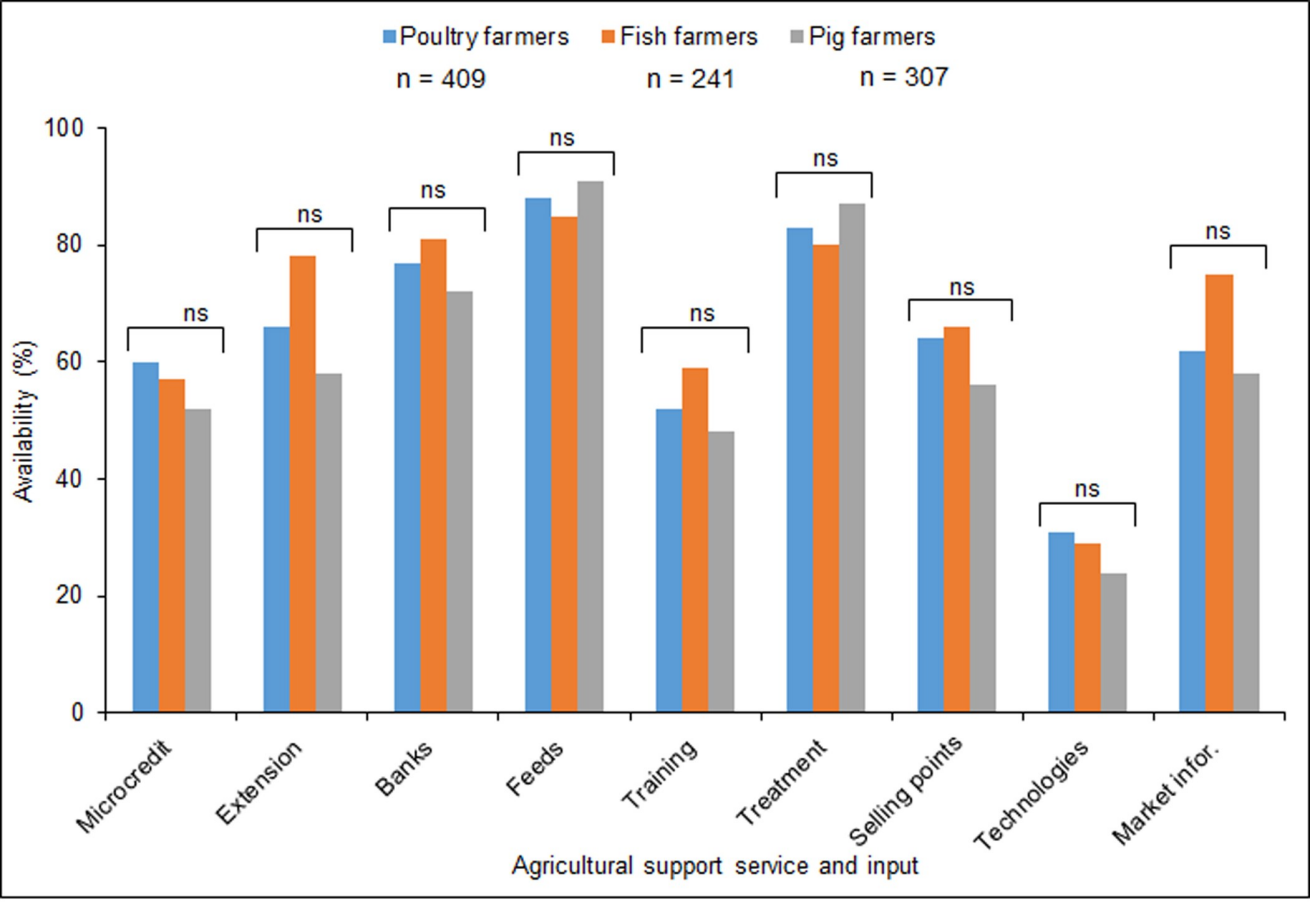
Percentage availability of agricultural support services and inputs to poultry, fish and pig farmers. Bars with “ns” are not significantly different, P < 0.05, Chi-squared test. Microcredit = availability of savings and credit cooperatives that provide saving and credit facilities at low interest rates; Extension = availability of agricultural extension services; Banks = availability of main stream banking services; Feeds = availability of commercial feeds and feed ingredients for the different livestock types; Training = availability of production education programs; Treatment = availability of vaccines and general disease control facilities; Selling points = selling points for poultry, fish and pig products; Technologies = availability of improved feeds, feeding, housing and general production methods; Market infor = availability of information regarding demand and supply of farm inputs and outputs.

Similar proportions of male and female farmers accepted cockroaches, housefly, BSF larvae, crickets, termites and grasshoppers as alternative feed components for poultry and fish production (Fig 2). The proportion of male pig farmers who accepted housefly and BSF larvae was significantly higher than for female pig farmers. However, similar proportions of male and female pig farmers accepted the other insect species investigated (Fig 2). The acceptance of termites was significantly higher compared to other insect species. The BSF larvae had the lowest acceptance, which differed significantly from other insect species for both male and female poultry, fish and pig farmers (S1 Table). Cockroaches, houseflies and crickets were similarly accepted as alternative feed ingredients for poultry fish and pig feed (S1 Table). Among support services and inputs, new technologies were the least available: ca 25–35% of farmers had access to this input (Fig 3).

### Willingness to pay for insect-based feeds (IBF) among poultry, fish and pig farmers

**Fig 4:**
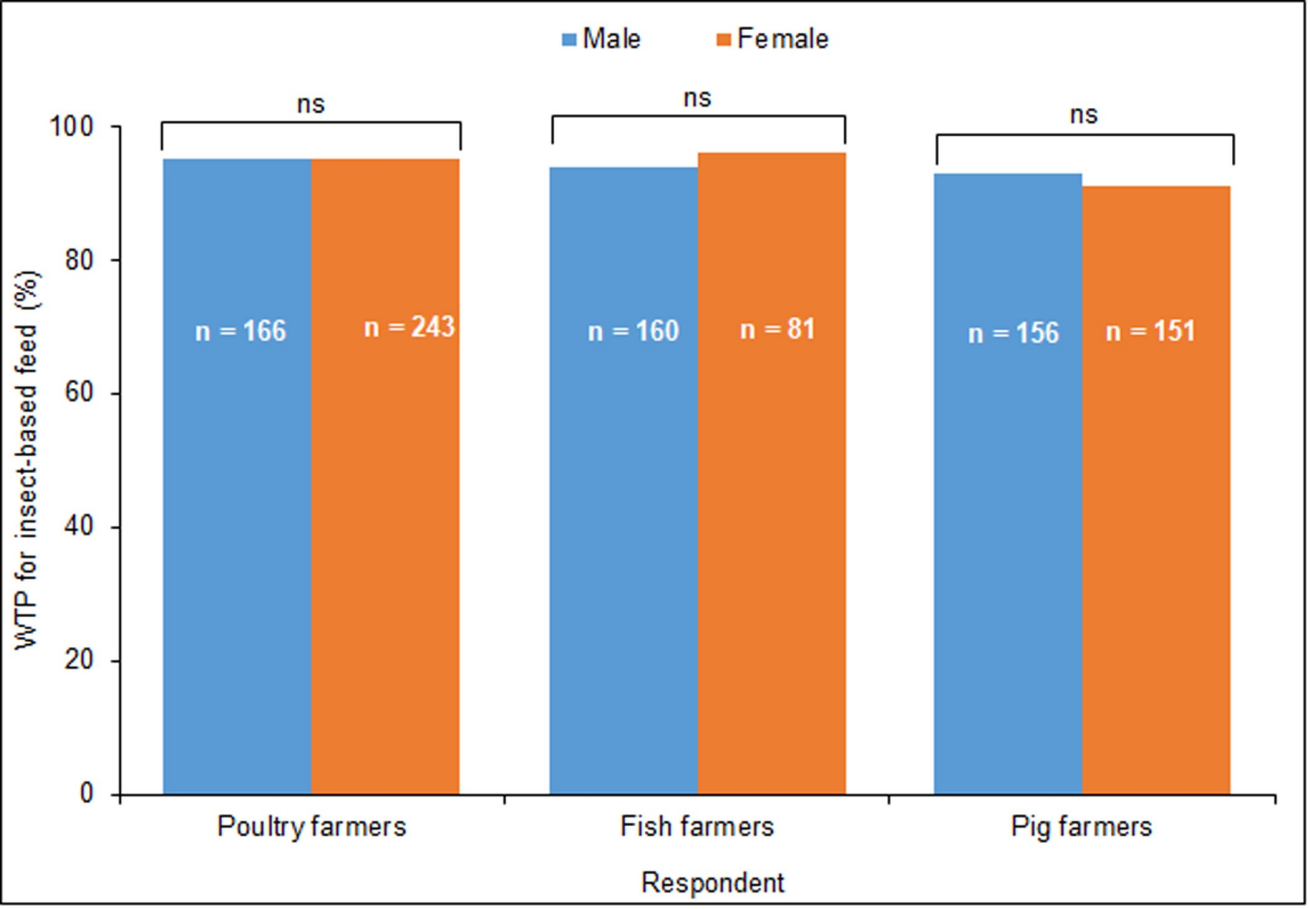
Percentage farmers willing to pay for insect-based feeds among male and female poultry, fish and pig farmers. Bars with “ns” are not significantly different for male and female respondents, P < 0.05, two-proportion z-test.

A total of 899 respondents were willing to pay for IBF, whereas 58 respondents were not, accounting for 94% and 6%, respectively. When asked if they would buy IBF (before the introduction of any bidding process), more than 90% of male and female poultry, fish and pig farmers responded positively (Fig 4).

**Fig 5:**
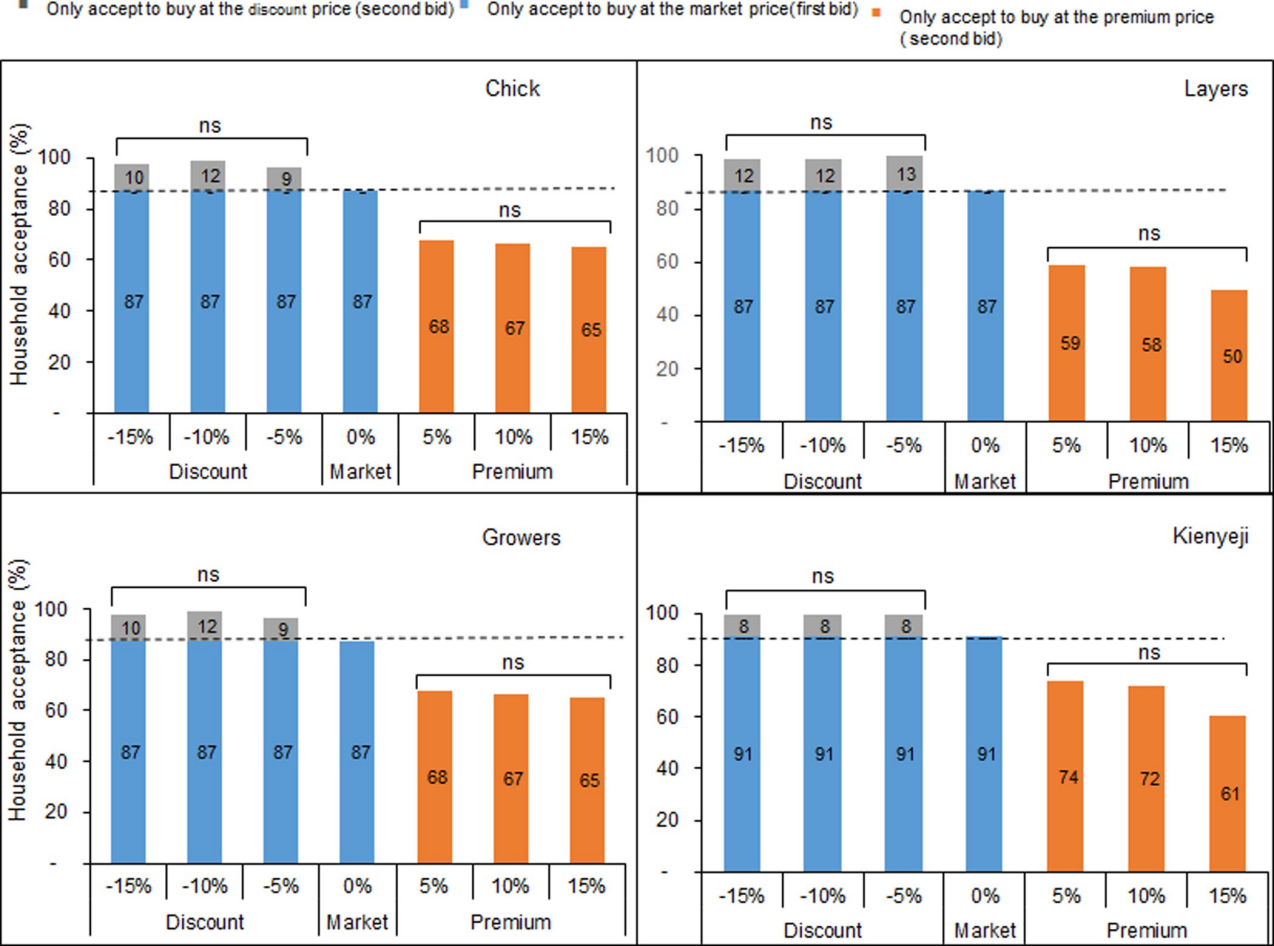
Effect of bid offers on willingness to pay (WTP) level among poultry farmers. The broken line indicates the market price. Bars with “ns” are not significantly different, P < 0.05, Chi-squared test. Chick mash = a ground form of feed fed to chicks aged 0–8 weeks. Growers = feed for birds aged 8–18 weeks. Layers = feed formulated for laying birds aged 19–76 weeks. Kienyeji = a ground form of feed for indigenous type of chicken commonly known as “Kienyeji”.

**Fig 6:**
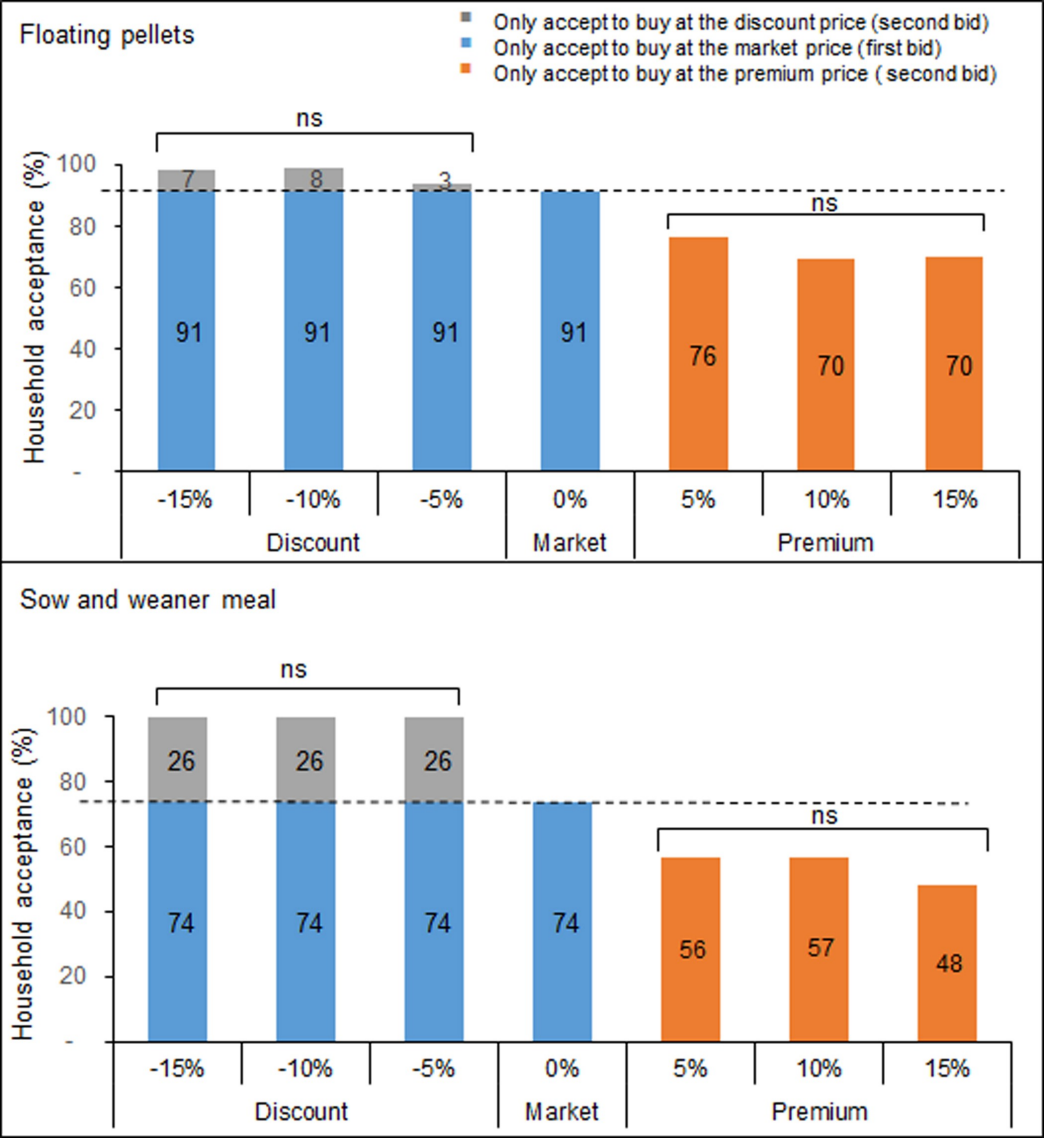
Effect of bid offers on willingness to pay (WTP) level among fish and pig farmers. The broken line indicates the market price. Bars with “ns” are not significantly different, P < 0.05, Chi-squared test. Floating pellets = feed that has been compressed and molded into pellets in a pellet mill and float on the surface of water when served to grower and finisher fish stages. Sow and weaner = Feed type for growing pigs up to 55 kg live body weight and adult breeding pigs. Pig finisher = Feed for pigs weighing over 55 kg live body weight.

For each animal category (poultry, fish and pig), more than 70% of the farmers were willing to buy the different feed types at the market price (S2, S3 and S4 Figs). WTP was high and ranged from 65–88% (S2, S3 and S4 Figs). Furthermore, 82–100%, 75–88% and 100% of all poultry, fish and pig farmers, respectively, were willing to buy at a discounted price (S2, S3 and S4 Figs). When the market price of feeds was reduced by 5–15%, most (96–100%) poultry farmers were willing to buy, whereas an increase in the market price resulted in a decrease in the percentage (50–74%) of poultry farmers willing to buy the different poultry feed types (Fig 5). The majority (94–99%) of the fish farmers were willing to buy floating pellets at a reduced price, but an increase in the market price resulted in a decrease in the percentage of farmers willing to buy at a premium price (Fig 6). Similarly, reducing the market price of sow and weaner feed by 5–15% resulted in all pig famers willing to buy at the reduced (discount) price (Fig 6). Furthermore, an increase in the price of feed reduced farmers’ WTP and less than 60% of the farmers accepted to buy sow and weaner feed at a premium price (Fig 6). At 15% increase, less than 50% of the pig farmers accepted to buy (Fig 6).

Farmers were willing to pay a premium price for IBF (Table 4). Poultry farmers were willing to pay Ksh 60–70 per kilogram of IBF, representing 16–57% increase from the benchmark price (market price) for the different poultry feed types. Kienyeji mash and broiler starter feeds had the highest and lowest percentage change, respectively for poultry farmers. Fish farmers had the lowest percentage change (12–28%) compared to the other farmers in the study. Pig farmers accepted to pay 30–70% higher prices for IBF (Table 4).

**Table 4:** Farmer’s willingness to pay (WTP), confidence intervals and mean price premium (percentage change) for insect-based feed.

	Feed type	WTP price (Ksh/kg)	95% Confidence Interval		Market price (Ksh/kg)	Standard error	Premium (%)
			Lower	Upper			
	Chick mash	70.05	65.06	75.04	48.92	1.04	43.19
	Growers mash	63.76	58.46	69.05	46.06	1.12	38.43
**Poultry**	Layers mash	57.92	53.76	62.08	44.04	1.19	31.52
	Kienyeji mash	58.47	51.54	65.41	37.34	1.21	56.59
	Broiler starter	71.11	66.98	75.23	61.32	1.91	15.97
	Broiler finisher	62.41	46.38	78.44	48.62	3.7	28.36
	Mash	101.92	84.59	119.26	91.12	9.98	11.85
**Fish**	Pellets	179.44	144.53	214.36	139.82	12.59	28.34
	Creep feed	121.17	23.86	266.19	49.38	10.04	70.49
**Pig**	Sow and weaner	53.47	47.47	59.47	35.69	1.42	49.82
	Pig finisher	52.83	45.51	60.15	40.65	3.48	29.96

Premium (%) = ((WTP price–Market price)/Market price) * 100, Ksh: Kenyan shillings. WTP = willingness to pay. Chick mash = a ground form of feed fed to chicks aged 0–8 weeks, Growers mash = feed for birds aged 8–18 weeks, Layers mash = feed for laying birds aged 19–76 weeks, Kienyeji mash = feed for indigenous type of chicken commonly known as “Kienyeji”. WTP = willingness to pay. Broiler starter and finisher = a protein-dense feed formulated to meet the dietary requirements of young broilers aged approximately 1–21 days and raised purposely for meat. Broiler finisher = feed formulated to meet the dietary requirements of broilers aged above 21 days. Mash = a finely ground feed formulated and used in moist form for farmed juvenile fish (fry), Floating pellets = finely ground feed that has been compressed and molded into pellets in a pellet mill and float on the surface of water when served to grower and finisher fish stages, Creep feed = high-nutrient feed designed to supplement nursing animals, Sow and weaner = Feed type for growing pigs up to 55 kilograms live body weight and adult breeding pigs, Pig finisher = Feed for pigs weighing over 55 kilograms live body weight.

### Factors influencing farmers’ willingness to pay (WTP) for insect-based feed

Explanatory variables were regressed with WTP for IBF among farmers for the different feed types within the animal categories (Tables 5 and 6). The regression results showed that ‘aware insects can be used as feeds, availability of extension and treatment services’ significantly and positively influenced WTP, whereas variables such as ‘make own poultry feed, and use of hired labour significantly and negatively influenced WTP among farmers using Kienyeji feed (Table 6). For famers using layers feed, use of conventional feeds and availability of training significantly and positively influenced WTP, whereas marital status, household size, distance to feed trader, awareness that insects can be used as poultry feed, availability of credit and market information and being in Kiambu or Nyeri relative to being in Uasin Gishu significantly and negatively influenced WTP (Table 6). For farmers using grower feed, use of conventional feeds and availability of treatment services significantly and positively influenced WTP (Table 6). For farmers using chick feeds, use of conventional feeds and availability of credit positively influenced WTP (Table 6).

**Table 5:** Regression results for factors influencing willingness to pay (WTP) for insect-based feeds among fish and pig farmers.

Variables	Fish Pellets			Mash			Sow and Weaner			Pig Finisher		
	95% confidence Interval			95% confidence Interval			95% confidence Interval			95% confidence Interval		
	Marginal Effects	Lower	Upper	Marginal Effects	Lower	Upper	Marginal Effects	Lower	Upper	Marginal Effects	Lower	Upper
Gender of the respondent (1 = Male, 0 = Female)	28.096	-16.366	72.559	-	-	-	-0.308	-12.045	11.430	-1.133	-13.261	10.994
Age (Years)	1.896	-7.779	11.571	-13.061**	-23.483	-2.639	0.781	-1.404	2.966	0.517	-2.670	3.705
Age squared	-0.026	-0.124	0.071	0.110**	0.015	0.206	-0.006	-0.029	0.017	0.001	-0.035	0.036
Education level (years)	4.215	-1.515	9.946	2.486	-0.920	5.891	-1.576	-3.599	0.446	-1.347	-3.391	0.697
Marital status (1 = Married, 0 = otherwise	6.808	-51.807	65.423	-5.266	-19.406	8.874	-3.794	-10.742	3.154	10.299	-12.946	33.543
Household Size (Number)	-6.923	-18.766	4.919	3.109	-1.621	7.838	-0.316	-3.088	2.456	-1.003	-4.664	2.658
Log of Income	14.825	-7.214	36.864	5.246	-3.465	13.957	3.617	-1.206	8.441	5.978	-1.341	13.296
Use conventional feeds (1 = Yes, 0 = No)	39.357	-9.243	87.957	-68.664***	-102.834	-34.495	-	-	-	-	-	-
Use commercial feeds (1 = Yes, 0 = No)	112.588**	18.869	206.307	-25.126	-59.919	9.666	-	-	-	-	-	-
Distance to feed trader (Km)	-0.167	-0.367	0.034	4.695**	0.579	8.811	-0.041	-0.219	0.137	0.780***	0.365	1.195
Ever used insects as feed (1 = Yes, 0 = No)	-70.283***	-115.053	-25.514	17.861	-7.796	43.517	-	-	-	-	-	-
Number of insects preferred (Count)	-3.226	-17.206	10.753	-2.665	-10.588	5.259	0.524	-2.509	3.558	-8.146**	-14.601	-1.691
Micro credit (1 = Yes, 0 = No)	-24.819	-71.121	21.483	30.218**	4.442	55.994	2.909	-7.271	13.089	-2.914	-14.662	8.834
Extension (1 = Yes, 0 = No)	52.158**	6.616	97.700	-46.171*	-92.756	0.414	4.802	-7.014	16.617	-12.597	-28.804	3.609
Agricultural inputs (1 = Yes, 0 = No)	0.900	-79.719	81.518				-4.599	-25.851	16.652	-4.747	-24.584	15.090
Treatment (1 = Yes, 0 = No)	12.341	-80.033	104.715	34.606**	2.110	67.101	-	-	-	-	-	-
Market information (1 = Yes, 0 = No)	-3.981	-61.777	53.814	-	-	-	-	-	-	-4.945	-20.097	10.207
Kiambu	-149.913***	-250.474	-49.352	98.407	-11272.210	11469.024	-4.986	-28.053	18.081	19.882	-8.790	48.554
Nyeri	-61.577	-138.702	15.548	-6.806	-52.479	38.866	-16.386	-40.462	7.691	11.664	-19.719	43.046
Kakamega	-8.464	-77.885	60.957	26.432	-8.843	61.707	12.331	-15.837	40.500	33.027**	1.598	64.457
Do you make own pig feed (1 = Yes, 0 = No)	-	-	-	-	-	-	-6.004	-17.823	5.815	-9.849	-34.431	14.733
New technologies (1 = Yes, 0 = No)	-	-	-	-	-	-	-1.068	-12.513	10.377	40.651***	14.299	67.003

Significance levels:

*** P < 0.01,

** P < 0.05,

* P < 0.1, logistic regression. (–) variable not included in the model. Floating pellets = finely ground feed that has been compressed and molded into pellets in a pellet mill and float on the surface of water when served to grower and finisher fish stages. Mash = a finely ground feed formulated and used in moist form for farmed juvenile fish (fry). For pig finisher = Feed for pigs weighing over 55 kilograms live body weight. Sow and weaner = Feed type for growing pigs up to 55 kilograms live body weight and adult breeding pigs. For Kienyeji = feed for indigenous type of chicken commonly known as “Kienyeji”. Layers = feed for laying birds aged 19–76 weeks. Growers = feed for birds aged 8–18 weeks. Chick = a ground form of feed fed to chicks aged between 0–8 weeks. WTP = willingness to pay.

**Table 6:** Regression results for factors influencing willingness to pay (WTP) for insect-based feeds among poultry farmers.

Variables	Kienyeji Mash			Chick Mash			Growers Mash			Layers Mash		
	95% confidence Interval			95% confidence Interval			95% confidence Interval			95% confidence Interval		
	Marginal Effects	Lower	Upper	Marginal Effects	Lower	Upper	Marginal Effects	Lower	Upper	Marginal Effects	Lower	Upper
Gender of the respondent (1 = male, 0 = female)	-6.521	-17.849	4.807	0.598	-9.216	10.411	-1.828	-12.856	9.200	3.792	-3.234	10.818
Age (Years)	-2.302	-6.757	2.153	-0.846	-3.459	1.768	-0.588	-3.027	1.851	-1.244	-2.857	0.370
Age squared	0.021	-0.025	0.068	0.010	-0.016	0.036	0.010	-0.016	0.036	0.012	-0.005	0.029
Education level (Years)	0.125	-1.274	1.525	-0.386	-1.654	0.882	-0.425	-1.968	1.119	-0.662	-1.651	0.328
Marital status (1 = Married, 0 otherwise)	-1.488	-6.268	3.291	2.126	-4.133	8.385	-2.079	-7.698	3.540	-4.929**	-8.891	-0.968
Household Size (Number)	0.507	-1.969	2.983	-0.300	-2.334	1.735	-1.662	-3.906	0.582	-3.843***	-5.677	-2.009
Log of income (Kshs)	3.255	-1.540	8.050	0.960	-2.941	4.860	0.685	-3.474	4.844	1.482	-1.369	4.332
Do use conventional feeds (1 = Yes, 0 = No)	8.839	-4.412	22.089	13.408**	1.472	25.343	9.906*	-1.858	21.671	9.634**	2.291	16.977
Do use commercial feeds	-11.892	-32.556	8.772	2.637	-17.589	22.864	-35.711	-7053.935	6982.512	-27.107	-4023.363	3969.149
Distance to feed trader (Km)	0.006	-0.141	0.154	0.014	-0.199	0.227	0.022	-0.210	0.255	-0.033**	-0.064	-0.002
Do you make own poultry feed (1 = Yes, 0 = No)	-19.630**	-35.937	-3.323	-	-	-	-	-	-	-	-	-
Aware feed on insects (1 = Yes, 0 = No)	17.521*	-2.597	37.639	0.574	-8.845	9.992	-	-	-	-9.781*	-19.711	0.149
Insect good source of feed (1 = Yes, 0 = No)	-14.198	-34.530	6.134	-	-	-	-	-	-	8.117	-2.154	18.388
Number owned growers	0.048	-0.098	0.195	-	-	-	0.045	-0.109	0.199	0.003	-0.035	0.040
Number owned chicks	-0.270	-0.811	0.271	-	-	-	0.246	-0.098	0.589	0.103	-0.153	0.360
Number of insects preferred (Count)	1.300	-1.926	4.526	1.532	-0.677	3.742	1.380	-1.386	4.147	1.022	-0.837	2.881
Micro credit (1 = Yes, 0 = No)	-3.667	-13.006	5.672	7.356*	-1.314	16.027	-3.773	-13.911	6.365	-7.532**	-14.485	-0.578
Extension (1 = Yes, 0 = No)	20.085***	7.447	32.723	0.709	-8.656	10.073	7.585	-4.289	19.460	-1.335	-8.388	5.719
Training (1 = Yes, 0 = No)	-9.623*	-20.409	1.164	-	-	-	-7.371	-19.347	4.606	7.757**	0.922	14.593
Treatment (1 = Yes, 0 = No)	12.044*	-0.964	25.051	-	-	-	10.394*	-1.897	22.685	-	-	-
Market information (1 = Yes, 0 = No)	3.735	-5.928	13.398	-	-	-	-0.202	-10.301	9.897	-6.729**	-13.185	-0.274
Kiambu	3.282	-14.323	20.886	-1.093	-21.276	19.090	-1.013	-17.314	15.289	-15.162***	-26.287	-4.037
Nyeri	-8.582	-20.468	3.303	-7.269	-19.361	4.824	-1.732	-15.986	12.522	-11.707*	-24.322	0.908
Kakamega	-2.214	-17.692	13.265	4.488	-5.777	14.753	9.127	-3.365	21.618	2.317	-7.712	12.345
Uses Family Labour (Poultry)	-22.639	-51.764	6.485	-5.462	-28.505	17.582	-22.395	-53.536	8.746	-14.725*	-29.950	0.500
Uses Hired Labour (Poultry)	-25.306***	-42.155	-8.457	1.200	-13.749	16.148	-3.761	-22.045	14.523	-2.455	-10.705	5.796

Significance levels:

*** P < 0.01,

** P < 0.05,

* P < 0.1, logistic regression. (–) variable not included in the model. For Kienyeji = feed for indigenous type of chicken commonly known as “Kienyeji”. Layers = feed for laying birds aged 19–76 weeks. Growers = feed for birds aged 8–18 weeks. Chick = a ground form of feed fed to chicks aged between 0–8 weeks. WTP = willingness to pay.

In fish production, WTP for IBF also varied for the different feed types (Table 4). The use of commercial feeds significantly and positively influenced WTP for farmers using floating pellets, whereas ‘ever used insects as feed, being in Kiambu relative to being in Uasin Gishu, significantly and negatively influenced WTP. For farmers using mash feed, farmers experience measured by the square of age, distance to the feed trader, availability of credit and treatment services significantly and positively influenced WTP whereas age, use of conventional feeds and availability of extension services significantly and negatively influenced WTP (Table 5).

In pig production, WTP was significantly and positively influenced by distance to the feed trader, being in Kakamega and availability of new technologies, whereas factors such as the number of insects preferred significantly and negatively influenced WTP among farmers using pig finisher feeds (Table 5). For farmers using sow and weaner feeds, WTP was not significantly affected by the variables (Table 5).

## Discussion

In most developing countries, agribusiness is an important component of the economy. Central to its sustainability are consumers’ attitudes and market acceptance of the products [41,44]. One way of ensuring this, is by assessing the hypothetical WTP prior to production [41,45]. The present study explored farmers’ knowledge of and attitudes towards insects as an alternative feed ingredient, the practice and utilization of local feed formulations, and their WTP for IBF. Socio-demographic characteristics of a household present the ability of the household to produce and consume goods. They affect the household’s access to and WTP for farm inputs [64]. The present study shows that farmers in the various regions are sufficiently educated and thus exposed to information which is important in decision making. In most peasant economies in developing countries, household labour is used to produce either for their own use or for the market [65]. In the present study, we recorded a mean household size of five, which indicates that family labour is available for production. In addition, farmers generally had a medium to high access to agricultural inputs and support services such as microcredit, extension services, banking services and agricultural inputs such as: feed, training, selling points for livestock products, new technologies and market information.

Information regarding farmers’ knowledge, attitude towards insects as feed and the practice of making and using their own feeds may be used in strategies geared towards introducing IBF. Our results show that Kenyan livestock producers are well aware of the potential of insects being used as a feed ingredient. This awareness probably provided farmers with the opportunity to develop a positive attitude. This positive attitude would promote the decision maker taking a risk regarding a novel input while a negative attitude discourages the decision maker from taking risk. This result is similar to previous reports that socio-economic characteristics and farmers’ knowledge affect interests in insect as feed [38]. The finding that farmers are well aware of the use of insects as feed ingredient with a positive attitude towards insects as a feed ingredient as well as the finding that farmers have already used insects to feed their animals, provides an enabling environment for implementing IBF.

A key finding in the present study is that farmers are willing to pay more for IBF than for major commercial feeds used in poultry, fish and pig production. This is consistent with the farmers’ high knowledge level and positive attitude towards insects as an alternative feed ingredient in this study and agrees with observations from studies in other countries [38]. This provides an indication of a potential market acceptance of IBF among farmers. The high percentage of farmers’ WTP recorded in this study is not surprising considering that insects have been part of the natural diet of poultry, fish and pigs in their natural environment [37].

The socio-demographic variables represent differential influences on farmers’ WTP in this study. The variable ‘age’ for example, negatively correlated with WTP for farmers using Kienyeji and floating pellets for poultry and fish, respectively, but showed a positive correlation with WTP for pig finisher feed. These results are in agreement with previous reports that the variable ‘age’ could either negatively or positively influence a farmer’s WTP for a new product [44, 50, 66]. Similar findings were obtained for variables such as ‘aware that insects can be used as feed’, which positively and negatively correlated with WTP for Kienyeji and layers feed, respectively.

It is worth noting that for poultry and pig, farmer age and acceptance of insects showed a significant correlation with WTP, in at least one of the feed types for each animal category. A negative correlation of ‘farmer age’ with WTP indicates that the younger the farmers, the more willing they are to pay for IBF for fish and vice versa [38]. This also agrees with other reports that younger people are more willing to try new products than older people [40]. This also suggests a greater potential adoption of insects as feed ingredient among young farmers compared to older farmers. Furthermore, acceptance of insects as feed ingredients generally showed a significant correlation with WTP. This means that an increased availability of the BSF would greatly influence farmers’ WTP for IBF.

We used household-based data and adopted the contingent valuation method [44] and the double bound-logit model to assess farmers’ WTP, WTP level and the factors that influence WTP, using market price as a benchmark price for IBF for poultry, fish and pig farmers in Kenya. Our results show that above 90% of the surveyed male and female farmers are willing to pay for IBF. The farmers are willing to pay at least 16%, 12% and 30% extra for IBF for poultry, fish and pigs, respectively. Female poultry farmers are significantly more aware that insects can be used as feed ingredients than males. More female poultry farmers have a positive attitude towards insects as feed than males. In one study for example, respondents expressed a strong negative attitude towards fish raised on IBF. When informed of the impacts of overfishing for farmed fish, the result showed a strong positive correlation, indicating that respondents’ knowledge can strongly affect acceptance and willingness to accept alternatives [34].

The present study is one of only few studies to assess producer opinion on insects as feed, and the first to document farmers’ knowledge, attitude towards insects as feed and their intentions to purchase IBF in sub-Saharan Africa. We conclude that farmers are willing to pay for IBF and understand the benefits of using IBF in animal production. Farmers’ WTP for IBF is a function of several socio-demographic factors, especially age, gender, awareness of insects as feed, acceptance of insect species, availability of agricultural extension services and market information. Therefore, such factors are crucial for designing policy strategies to ensure effective adoption of IBF in Kenya.

Despite the sparse body of research related to the use of insects as ingredients in animal feeds, producer acceptance will not be a barrier towards the development of the insect protein industry for feed. The implication of our study further supports previous on-station insect-based feeding trials on poultry, pig and fish with positive results that were comparable or superior to that of conventional feeds (Chia et al. in press). Therefore, feed companies can anticipant using insects as alternative high-quality protein ingredients to partially or completely replace the expensive and scarce fish and soybean meals in poultry, fish and pig feeds. However, further research is urgently needed to optimize insect meal inclusions in commercial feeds for enhanced livestock and fish production. The present study further supports previous observations given that most of the farmers in the study areas are willing to pay for IBF. The International Centre of Insect Physiology and Ecology (*icipe*) and its partners have optimized and established mass rearing technologies for edible species that could be used in animal feeds, documented nutritional profile of these insects, standardized safe processing, post-harvest handling and packaging technologies, developed and tested over 35 insect-based feed formulations for poultry, fish, and pigs. These outcomes have led to the development and gazettement of the first insect-based feed standards in Kenya and Uganda allowing the use of dried insect products as protein additives in compounded animal feeds–a first of its kind on the African continent after that of the United State of America. The creation of an enabling environment for integrating insect-based meals in compounded animal feeds in Kenya and Uganda, has contributed to opening new markets and opportunities for commercialization of insect-protein based products. Also, there is enormous demand from African governments for their wide promotion and scaling for impacts, especially to enhance job opportunities for youths and women across the feed value chain. This can be achieved through awareness creation, capacity building, dissemination of the insect-based feed enterprises and strengthening stakeholders and market linkages for sustainability and improved livelihoods.

Although, there is no capacity in Kenya to sustainably produce insect-based feeds to meet the market demand, there are indications that rolling-out insect-based feed protein technologies would have a huge potential for improving poultry, pig and fish production, employment opportunities and livelihoods of the populations in the various Counties. Furthermore, these results present an excellent opportunity for innovative and sustainable use of resources through insect rearing and minimizes the pressure on the agricultural land and marine resources. Our findings provide the first insight into the market opportunities of including insects in the animal feed value chain in Kenya, particularly following the recent authorization of the use of insects in animal feed by the Kenyan government [67].

To enhance farmer uptake of the innovative technology and WTP for more sustainable and readily available alternatives such as IBF, improvements in extension support services are of paramount importance [4, 39]. We therefore recommend the following: First, there is a need to increase farmer’s knowledge on the nutritional value of insects, especially the BSF, as well as their use as alternative feed ingredients. From the empirical results of this study, awareness and acceptance of insect species significantly influence farmers’ WTP. Developing local insect production systems can increase availability of insect meal and, thus promote local IBF production. In the present study for instance, ‘‘distance to feed trader” had a significant influence on WTP. This means that the closer the feed trader is to the farmer, the higher the probability that the farmer will be willing to buy the feed and vice versa. Second, our results indicate that the use of commercial feeds and availability of extension significantly and positively influence WTP among fish farmers for floating pellets. This means that an increase in these variables leads to an increase in WTP. So, engaging feed millers and traders and training them with regard to the advantages of insect meal in animal feed, can promote inclusion of insects in commercial feed without affecting demand. Finally, creating linkages between farmers and the markets will further enhance utilization of innovative and locally available feed resources such as insect meal among fish and livestock farmers in Kenya.

## Supporting information

S1 FigDouble-bound dichotomous choice contingent valuation methods bid sequence.$Bid_{i}^{H}=$ the second bid, which is an amount greater than the first bid (*Bid*_*i*_); $Bid_{i}^{L}=$ the second bid, which is an amount smaller than the first bid if the individual response is “no” to the first bid.(TIFF)

S2 Fig Percentage farmers willing to pay for insect-based poultry feeds at market price, discount price and premium price. Bars with “ns” are not significantly different, P < 0.05, Chi-squared test. Chick mash = a ground form of feed fed to chicks aged 0–8 weeks. Growers mash = ground form of feed for birds aged 8–18 weeks. Layers mash = ground form of feed for laying birds aged 19–76 weeks. Broiler starter = a protein-dense feed formulated to meet the dietary requirements of young broilers aged approximately 1–21 days and are raised purposely for meat. Kienyeji mash = a ground form of feed for indigenous type of chicken commonly known as “Kienyeji”.
(TIF)

S3 Fig Percentage farmers willing to pay for insect-based fish feed at market, discount and premium price. Bars with an asterisk are significantly different for mash and floating pellets, P < 0.05, z-test. Bars with “ns” are not significantly different, P < 0.05, two-proportion z-test. Mash = a finely ground feed formulated and used in moist form for farmed juvenile fish. Floating pellets = finely ground feed that has been compressed and molded into pellets in a pellet mill and float on the surface of water when served to grower and finisher fish stages.
(TIF)

S4 Fig Farmers’ willingness to pay (WTP) for insect-based pig feed at market, discount and premium price. Bars with “ns” are not significantly different, P < 0.05, Chi-squared test. Sow and weaner = Feed type for growing pigs up to 55 kg live body weight and adult breeding pigs. Pig finisher = Feed for pigs weighing over 55 kg live body weight.
(TIF)

S1 Table Comparison of farmers’ acceptance of different insect species as feed ingredients. (DOCX)

S1 Raw data Global positioning system coordinates of surveyed locations. (XLS)

S2 Raw data Socioeconomic field survey questionnaire data. (XLSX)
