# Supplementary figures and images for: Smallholder farmers’ knowledge and willingness to pay for insect-based feeds in Kenya
Source: PLoS One. 2020 Mar 25;15(3):e0230552. doi: 10.1371/journal.pone.0230552 (PMC7094868; doi:10.1371/journal.pone.0230552)

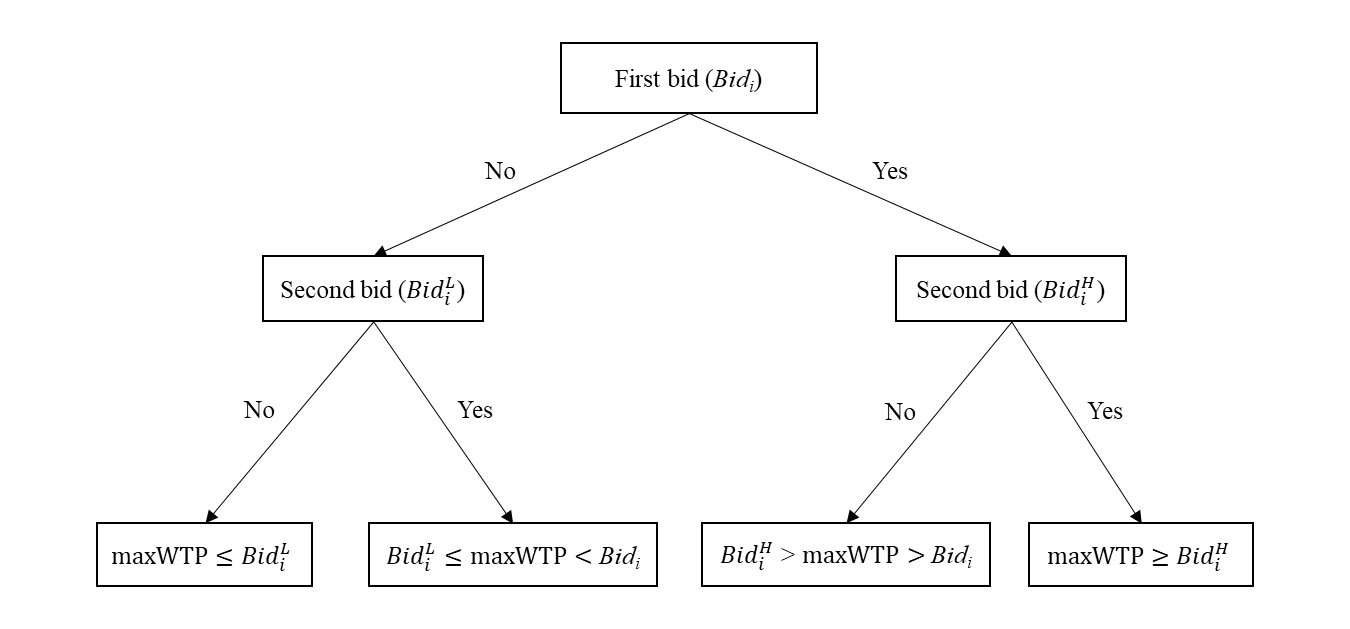

Supplement: S1 Fig — BidiH= the second bid, which is an amount greater than the first bid (Bidi); BidiL= the second bid, which is an amount smaller than the first bid if the individual response is “no” to the first bid. (TIFF) [file pone.0230552.s001.tiff]

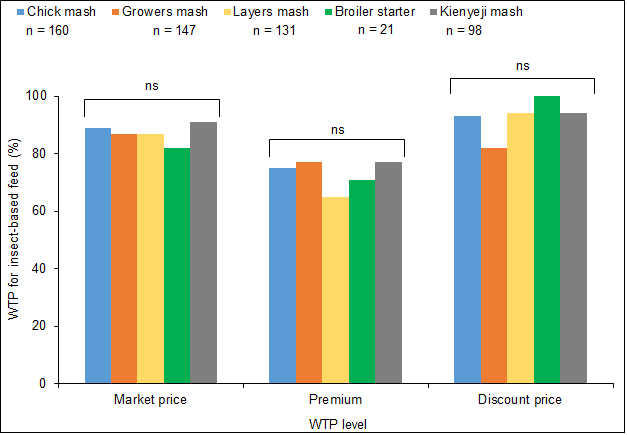

Supplement: S2 Fig — Bars with “ns” are not significantly different, P < 0.05, Chi-squared test. Chick mash = a ground form of feed fed to chicks aged 0–8 weeks. Growers mash = ground form of feed for birds aged 8–18 weeks. Layers mash = ground form of feed for laying birds aged 19–76 weeks. Broiler starter = a protein-dense feed formulated to meet the dietary requirements of young broilers aged approximately 1–21 days and are raised purposely for meat. Kienyeji mash = a ground form of feed for indigenous type of chicken commonly known as “Kienyeji”. (TIF) [file pone.0230552.s002.tif]

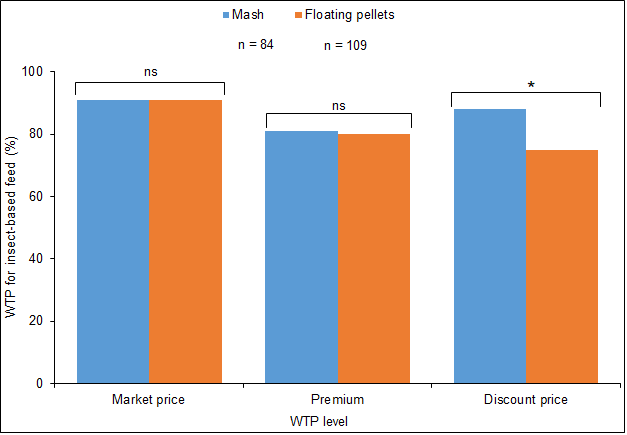

Supplement: S3 Fig — Bars with an asterisk are significantly different for mash and floating pellets, P < 0.05, z-test. Bars with “ns” are not significantly different, P < 0.05, two-proportion z-test. Mash = a finely ground feed formulated and used in moist form for farmed juvenile fish. Floating pellets = finely ground feed that has been compressed and molded into pellets in a pellet mill and float on the surface of water when served to grower and finisher fish stages. (TIF) [file pone.0230552.s003.tif]

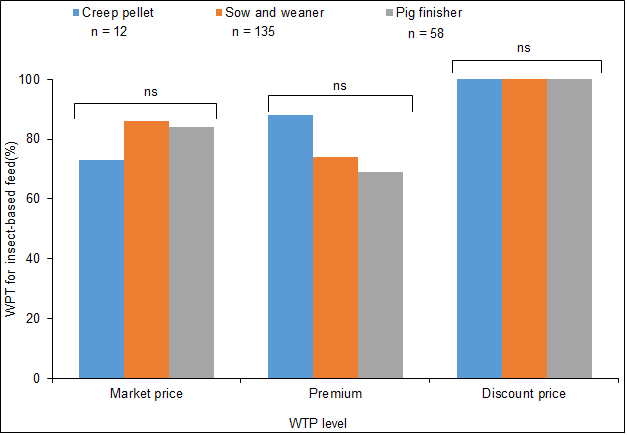

Supplement: S4 Fig — Bars with “ns” are not significantly different, P < 0.05, Chi-squared test. Sow and weaner = Feed type for growing pigs up to 55 kg live body weight and adult breeding pigs. Pig finisher = Feed for pigs weighing over 55 kg live body weight. (TIF) [file pone.0230552.s004.tif]
