# Supplementary material for: Smallholder farmers’ knowledge and willingness to pay for insect-based feeds in Kenya
Source: PLoS One. 2020 Mar 25;15(3):e0230552. doi: 10.1371/journal.pone.0230552 (PMC7094868; doi:10.1371/journal.pone.0230552)
Supplement: S1 Table — (DOCX) [file pone.0230552.s005.docx]

**Table S1:** Comparison of farmers’ acceptance of different insect species as feed ingredients

| Comparison | Poultry farmers | | | | Fish farmers | | | | Pig farmers | | | |
| --- | --- | --- | --- | --- | --- | --- | --- | --- | --- | --- | --- | --- |
|  | Male | | Female | | Male | | Female | | Male | | Female | |
|  | χ2 | Sig. | χ2 | Sig. | χ2 | Sig. | χ2 | Sig. | χ2 | Sig. | χ2 | Sig. |
| Cockroach vs Housefly | 1.75 | ns | 3.51 | ns | 0.14 | ns | 0 | ns | 1.64 | ns | 0.03 | ns |
| Cockroach vs BSF | 13.24 | *** | 30.32 | *** | 18.56 | *** | 14.51 | *** | 9.0 | ** | 20.55 | *** |
| Cockroach vs Termites | 36.63 | *** | 44.94 | *** | 27.69 | *** | 10.18 | ** | 18.28 | *** | 15.38 | *** |
| Cockroach vs Crickets | 5.31 | * | 0.85 | ns | 0.32 | ns | 0.28 | ns | 1.20 | ns | 0.79 | ns |
| Cockroach vs Grasshopper | 8.49 | ** | 8.16 | ** | 2.48 | ns | 5.50 | * | 9.15 | ** | 5.55 | * |
| Housefly vs BSF | 24.25 | *** | 53.25 | *** | 15.54 | *** | 14.51 | *** | 18.10 | *** | 19.04 | *** |
| Housefly vs Termites | 23.34 | *** | 24.55 | *** | 31.42 | *** | 10.18 | ** | 9.21 | ** | 16.74 | *** |
| Housefly vs Crickets | 0.98 | ns | 0.91 | ns | 0.04 | ns | 0.28 | ns | 0.04 | ns | 1.13 | ns |
| Housefly vs Grasshopper | 2.57 | ns | 0.98 | ns | 3.80 | ns | 5.50 | * | 3.09 | ns | 6.40 | * |
| BSF vs Termites | 86.77 | *** | 136.1 | *** | 83.10 | *** | 44.67 | *** | 50.46 | *** | 67.31 | *** |
| BSF vs Crickets | 34.39 | *** | 40.89 | *** | 14.14 | *** | 10.88 | ** | 16.59 | *** | 29.0 | *** |
| BSF vs Grasshopper | 41.48 | *** | 67.54 | *** | 33.76 | *** | 35.49 | *** | 35.14 | *** | 45.82 | *** |
| Termites vs Crickets | 15.24 | *** | 34.2 | *** | 33.34 | *** | 13.60 | *** | 10.35 | ** | 9.36 | ** |
| Termites vs Grasshopper | 10.99 | ** | 16.1 | *** | 14.47 | *** | 0.81 | ns | 1.69 | ns | 2.57 | ns |
| Crickets vs Grasshopper | 0.38 | ns | 3.77 | ns | 4.55 | * | 8.17 | ** | 3.78 | ns | 2.18 | ns |

Significance (sig.) level: *** P < 0.001; ** P < 0.01; * P < 0.05; ns = not significant; chi-squared test
